# Graphitic Carbon Nitride as Visible-Light Photocatalyst Boosting Ozonation in Wastewater Treatment

**DOI:** 10.3390/nano12193494

**Published:** 2022-10-06

**Authors:** Amarajothi Dhakshinamoorthy, Antón López-Francés, Sergio Navalon, Hermenegildo Garcia

**Affiliations:** 1 Department of Chemistry, Universitat Politècnica de València, C/Camino de Vera, s/n, 46022 Valencia, Spain; 2 School of Chemistry, Madurai Kamaraj University, Madurai 625021, Tamil Nadu, India; 3 Instituto Universitario de Tecnología Química, Consejo Superior de Investigaciones Científicas-Universitat Politècnica de València, Universitat Politècnica de València, Av. De los Naranjos s/n, 46022 Valencia, Spain

**Keywords:** carbon nitride, organic pollutants, ozone, photocatalysis, visible light

## Abstract

Light can boost ozone efficiency in advanced oxidation processes (AOPs), either by direct ozone photolysis with UV light or by using a photocatalyst that can be excited with UV-Vis or solar light. The present review summarizes literature data on the combination of ozone and the g-C_3_N_4_ photocatalyst for the degradation of probe molecules in water, including oxalic, p-hydroxybenzoic and oxamic acids as well as ciprofloxacin and parabens. g-C_3_N_4_ is a metal-free visible-light photocatalyst based on abundant elements that establishes a synergistic effect with ozone, the efficiency of the combination of the photocatalysis and ozonation being higher than the sum of the two treatments independently. Available data indicate that this synergy derives from the higher efficiency in the generation of hydroxyl radicals due to the efficient electron quenching by O_3_ of photogenerated conduction band electrons in the g-C_3_N_4_ photocatalyst. Given the wide use of ozonizers in water treatment, it is proposed that their implementation with g-C_3_N_4_ photocatalysis could also boost ozone efficiency in the AOPs of real waste waters.

## 1. Introduction

Advanced oxidation processes (AOPs) are well established treatments for wastewater remediation [1,2,3,4,5,6,7,8,9,10]. In the AOP, reactive oxygen species (ROS) are generated from oxygen or oxidizing agents by chemical, photophysical, electrochemical or any other means [11,12,13]. One of the most powerful AOP treatments uses ozone (O_3_) as a precursor of ROS and light to promote O_3_ conversion [14,15,16]. Since O_3_ absorbs in the UV region [17,18,19], the direct irradiation of O_3_ requires artificial light from lamps, thus making the whole process more costly. One improvement of this AOP based on O_3_ is the use of a photocatalyst that opens the possibility to use visible and even natural sunlight [20,21,22,23].

**Scheme 1:**
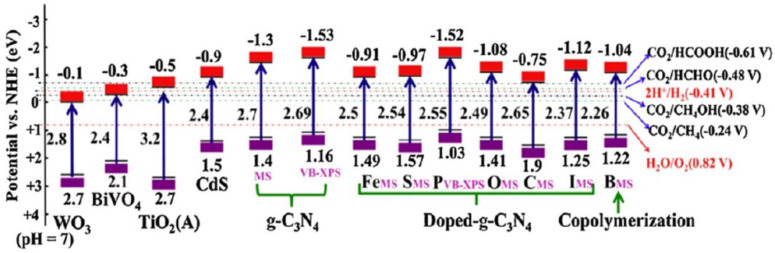
Conduction and valence band energy values of different types of g-C_3_N_4_ samples. Reproduced with permission from Ref. [30]. Copyright 2017 Elsevier.

Although g-C_3_N_4_ possesses similar structure to graphite, g-C_3_N_4_ exhibits a stacked 2D structure of sheets ideally consisting of *s*-tris triazine units condensed by tertiary nitrogen atoms. These layers interact by van der Waals forces, thus imparting this material with high thermal and chemical stability. The electronic structure of this material has a band gap of about E_g_~2.7 eV, thus finding applications in various fields, including the heterogeneous catalysis and photodegradation of organic pollutants [24,25,26,27,28,29]. The energy values of the conduction band and valence band are −1.1 and +1.6 eV, respectively, which are suitable to perform various redox reactions (Scheme 1). Some of the interesting features of g-C_3_N_4_ compared to other conventional photocatalysts are that g-C_3_N_4_ is a metal-free solid possessing solely carbon and nitrogen, which are highly abundant in earth, cost-effective, environmentally benign and highly safe, visible-light photoresponse and suitable band energy alignment. These interesting factors encouraged researchers to develop photocatalysts based on g-C_3_N_4_ for the degradation of organic pollutants without the use of transition metals.

**Scheme 2:**
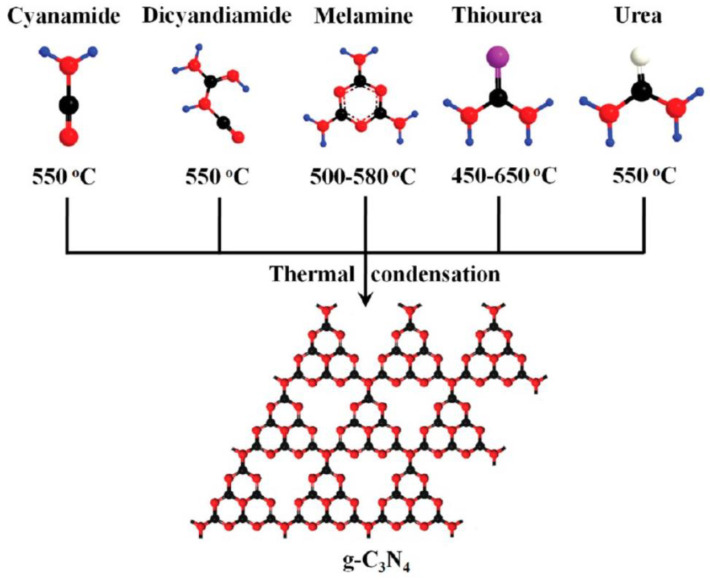
Schematic illustration of main routes for synthesis of g-C_3_N_4_ by condensation of cyanamide, dicyandiamide, melamine, thiourea, and urea. Reproduced with permission from Ref. [44]. Copyright 2015 Wiley.

Preparation of g-C_3_N_4_ is always a challenging process and many synthetic methods have been reported in the literature [31,32,33]. A variety of g-C_3_N_4_ structures have been prepared by employing liquid-based approaches through shaping and casting to obtain large surface-area solid; however, these approaches employ toxic chemicals [34,35]. One of the common strategies to prepare g-C_3_N_4_ is through the solid-state reaction from cyanuric chloride and/or, calcium cyanide, lithium azide, or melamine [36,37,38]. In recent years, the thermal decomposition of single precursors such as cyanamide [39], dicyanamide [40], melamine [41], thiourea [42], or urea [43] has also resulted in high-quality and low-defect density g-C_3_N_4_ materials (Scheme 2).

The morphology of pristine g-C_3_N_4_ can significantly be altered due to the presence of acidic and C−N bond in its structure. Thinner nanosheets of g-C_3_N_4_ can also be prepared retaining their characteristic structural features by exfoliation and treatment with inorganic acids. Transition metal carbides can also be used to prepare thin g-C_3_N_4_ nanosheets, but these metal carbides can also be an active site in the photocatalytic reaction. Porosity has been tried to introduce in g-C_3_N_4_ by making use of the bubble effect, but it is still a challenge to regulate pore size. Overall, the morphology of g-C_3_N_4_ can easily be designed and controlled for a required application with the advantages of easy recovery, high mechanical resistance, and good photocatalytic performance.

As it will be discussed in different examples, the generation of ^•^OH is highly prominent upon visible-light irradiation of g-C_3_N_4_ in the presence of O_3_ due to the synergistic effect summarized in Equations (1)–(4). In the Vis/O_3_/g-C_3_N_4_ photocatalyst, g-C_3_N_4_ can absorb the photons under the visible-light irritation to generate e^-^ upon CB and holes (h^+^) on the VB (Equation (1)). The CB potential of g-C_3_N_4_ can be as low as −0.78 V (versus SCE at pH 7), which significantly facilitates CB electron capture by O_3_. As a result, an ozonide radical (^•^O_3_^−^) is produced (Equation (2)), and it quickly protonated in the medium to generate a HO_3_^•^ radical (Equation (3)). This trioxide radical easily decomposes into ^•^OH (Equation (4)). The operation of these equations requires the combination of g-C_3_N_4_ and O_3_ and would not take place in the absence of one of these reagents.

g-C_3_N_4_ + Vis → e^−^ + h^+^ (1)

(2) $$O_{3}+e^{-}\to \cdot O_{3}^{-}$$

(3) $$\cdot O_{3}^{-}+H^{+}\to {\mathrm{HO}}_{3}^{\cdot }$$

(4) $${\mathrm{HO}}_{3}^{\cdot }\to O_{2}+{\mathrm{HO}}^{\cdot }$$

Considering the recent progress made on wastewater treatment through the AOP [45,46], the present review focuses on the AOP combining O_3_ as the oxidizing reagent and graphitic carbon nitride (g-C_3_N_4_) without any metal as the photocatalyst. However, readers are directed to refer the catalytic activity of metal-doped TiO_2_ or Ag-ZnO photocatalysts for the degradation of pollutants [47,48]. The purpose of this review is to show that g-C_3_N_4_, in the absence of any precious metal or even any other transition metal, is a very efficient visible-light photocatalyst to activate O_3_ generating ROS. Among the various possible ROS, hydroxyl radicals (^•^OH) are the most powerful and aggressive species [49,50,51,52], since they have a high oxidation potential. They are also a strong electrophilic species and are able to abstract a hydrogen from virtually any C-H bond, generating carbon-centered radicals [53]. It will be shown in this review that the combination of O_3_ and a photocatalyst such as g-C_3_N_4_ is a general method to produce high fluxes of ^•^OH and, therefore, is a very powerful AOP treatment.

In many cases, model molecules have been used to evaluate and demonstrate the advantage of combining the g-C_3_N_4_ photocatalyst with visible light and O_3_. The present review is organized according to the probe used to demonstrate the efficiency of combining the g-C_3_N_4_ photocatalyst and O_3_. Oxalic acid (OA) as well as other reluctant organic pollutants that are selectively degraded by ^•^OH are the favorite probes to show the synergy between the g-C_3_N_4_ photocatalyst and O_3_. The last section summarizes the main achievement reached so far with the g-C_3_N_4_ photocatalyst and ozonation and provides our prospects for future developments in this field.

## 2. Oxalic Acid (OA)

**Figure 1:**
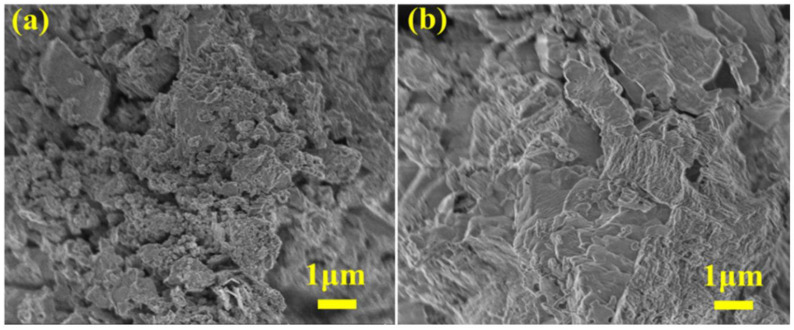
FESEM images of GCN-T (**a**) and GCN-D (**b**). Reproduced with permission from Ref. [54]. Copyright 2016 Elsevier.

**Figure 2:**
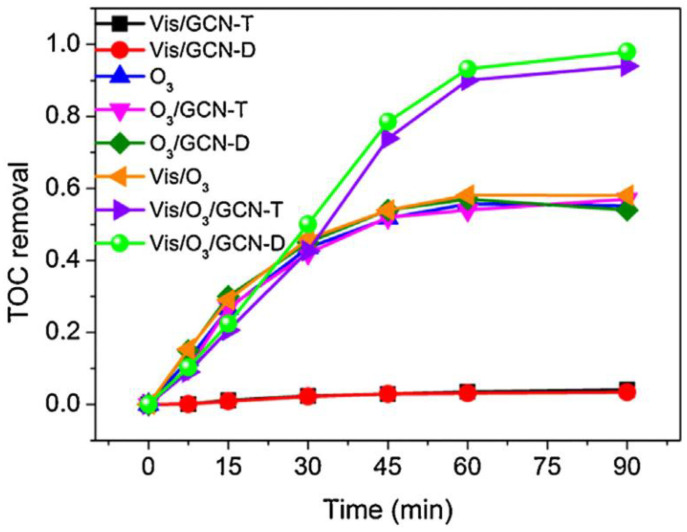
TOC removal of PHBA degradation under ozonation or irradiation under various conditions (gas flow rate: 100 mL/min; O_3_ concentration: 30 mg/L; light intensity: 365 mW/cm^2^; initial volume of solution: 400 mL; initial PHBA concentration: 40 mg/L; catalyst dosage: 1 g/L). Reproduced with permission from Ref. [54]. Copyright 2016 Elsevier.

In one of the studies showing the activity to generate ^•^OH radicals, bulk g-C_3_N_4_ was synthesized either from thiourea (GCN-T) or dicyandiamide (GCN-D), respectively. Figure 1 provides SEM images of the two GCN samples. The photocatalytic performance of these solids was tested in the mineralization of OA and p-hydroxybenzoic acid (PHBA, Figure 2) under UV and visible-light irradiation [54]. A synergy between photocatalysis by g-C_3_N_4_ under visible light and ozonation was found. Under the optimized reaction conditions, the rate constant observed for OA removal using Vis/O_3_/GCN-D was 20.6 times higher than the sum of that in Vis/GCN-D and ozonation. On the other hand, TOC removal of PHBA with Vis/O_3_/GCN-D was 98%, which is about 39.3% higher compared to the sum of the value observed with Vis/GCN-D and ozonation. Interestingly, the Vis/O_3_/GCN-D photocatalytic system showed stronger oxidizing capacity than UV/O_3_/GCN-D for OA degradation with the same light intensity. The inferior activity of UV/O_3_/GCN-D was proposed to be due to the partial direct irradiation of ozone that competes with ozone quenching of the photoinduced electrons on GCN that is the only operating process under visible-light irradiation. Consequently, the amount of produced ^•^OH decreased with UV in comparison to visible light.

**Figure 3:**
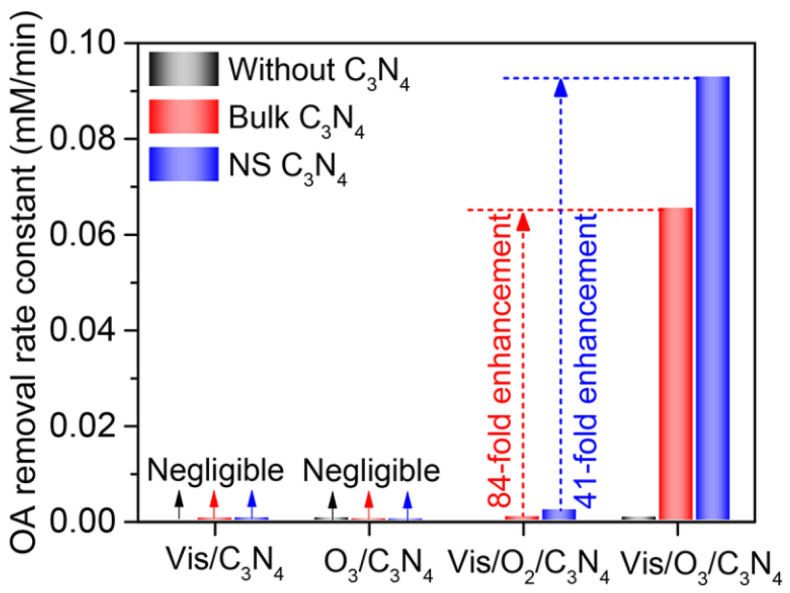
OA degradation rate constants upon irradiation of OA aqueous solutions in the presence of C_3_N_4_ under various conditions. Reproduced with permission from Ref. [55]. Copyright 2017 American Chemical Society.

**Figure 4:**
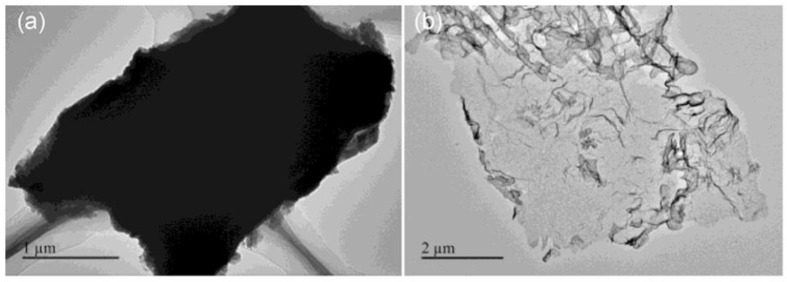
FETEM images of (**a**) bulk C_3_N_4_ and (**b**) NS C_3_N_4_. Reproduced with permission from Ref. [55]. Copyright 2017 American Chemical Society.

Recently, the photocatalytic generation of charge separation with the appearance of electrons and holes in C_3_N_4_ and their trapping by dissolved O_2_ and O_3_, as well as ROS evolution, was experimentally determined by spin-trapping upon feeding O_3_ into the Vis/O_2_/C_3_N_4_ photocatalytic system in aqueous media [55]. EPR measurements showed that a gas mixture of O_3_ (2.1 mol %)−O_2_ (97.9 mol %) can facilely trap about double to triple the amount of conduction band (CB)-e^−^ in an aqueous C_3_N_4_ suspension irradiated by visible light compared to pure O_2_. This is mainly due to the much higher redox potential and water solubility of O_3_ in comparison to O_2_. The capture of CB-e^−^ by O_2_ forms ^•^O_2_^−^, which later converted to ^•^OH through the H_2_O_2_-mediated consecutive three-electron-reduction pathway. Quantification of the EPR signal has shown a 17-fold enhancement in the formation of ^•^OH (characterized by the DMPO-OH adduct) and an 84-fold increase in the rate of OA mineralization when 2.1 mol % O_3_ is introduced in the Vis/O_2_(O_3_)/bulk C_3_N_4_ photocatalytic system (Figure 3). Interestingly, the rate constant was further increased by a factor of 41 when bulk C_3_N_4_ is exfoliated to the nanosheet (NS) C_3_N_4_ form (Figure 4). These results indicate that the use of NS C_3_N_4_ exhibits superior performance compared to bulk C_3_N_4_ due to its high surface area and upshifted CB edge, which favors more CB-e^−^ to be trapped by dissolved O_3_ and O_2_ and the formation of higher ^•^OH yield in photocatalytic ozonation compared to bulk C_3_N_4_.

**Figure 5:**
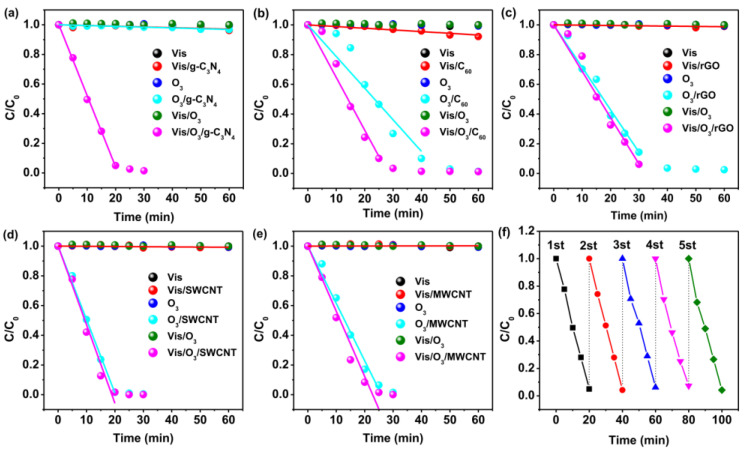
OA degradation upon ozonation and irradiation under various conditions in the presence of g-C_3_N_4_ (**a**), C60 (**b**), rGO (**c**), SWCNT (**d**) and MWCNT (**e**); Repeated photocatalytic ozonation for OA degradation over 0.1 g/L g-C_3_N_4_ (**f**) in five cycles. Reproduced with permission from Ref. [56]. Copyright 2016 Elsevier.

In another report, systematic studies were performed with a series of dimension-structured nanocarbons as the visible-light photocatalysts in the presence of O_3_ as the metal-free AOP catalyst for water disinfection [56]. Single-walled carbon nanotube (SWCNT), multi-walled carbon nanotube (MWCNT), reduced graphene oxide (rGO) and fullerene (C60) exhibited superior catalytic performance in catalytic ozonation, while g-C_3_N_4_ and C60 outperformed in the visible light-O_3_ coupled photocatalytic process. The results are presented in Figure 5. The coupling coefficient of visible light with ozone on g-C_3_N_4_ measuring the synergy arising from the combination of ozonation and photocatalysis in comparison with the sum of the separate treatments was found to be as 95.8. Both g-C_3_N_4_ and C60 promoted the synergism between the visible-light photocatalysis and O_3_ in the generation of ^•^OH radicals for the efficient removal of OA compared to O_3_/nanocarbon (SWCNT, MWCNT and rGO) or even benchmark photocatalysts such as Vis/O_3_/metal oxides (WO_3_ and TiO_2_). Among these carbon materials, the superior activity of g-C_3_N_4_ is due to the narrow bandgap and upshifted CB minimum of g-C_3_N_4_ for the visible-light photocatalytic ozonation.

**Figure 6:**
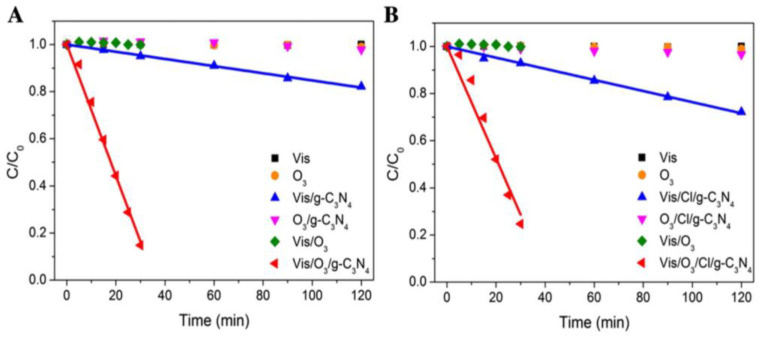
Degradation of OA by various oxidation processes upon g-C_3_N_4_ (**A**) and Cl/g-C_3_N_4_ (**B**). Reproduced with permission from Ref. [57]. Copyright 2015 Elsevier.

In another study, the coupling of g-C_3_N_4_ or chlorine modified g-C_3_N_4_ (Cl/g-C_3_N_4_) photocatalysts with ozonation was employed as an effective strategy for the mineralization of OA under a visible-light irradiation condition [57]. The use of g-C_3_N_4_ and Cl/g-C_3_N_4_ was able to trigger a synergy between photocatalysis and ozonation with a coupling coefficient of 17.8 and 9.9, respectively, compared to the sum of the OA degradation by the two treatments separately (Figure 6). Further, the combination of CB electrons and ozone effectively promoted surface-charge separation of g-C_3_N_4_ and self-decomposition of ozone to ^•^OH in a much higher selectivity. The experimental results indicated that ^•^OH is primarily responsible for the mineralization of OA in Vis/O_3_/g-C_3_N_4_, while in comparison ^•^O_2_^−^ and other ROS are more important than ^•^OH radicals in the g-C_3_N_4_ photocatalytic mineralization of OA. The removal efficiency of OA was 28% with Cl/g-C_3_N_4_ after 120 min under visible-light irradiation, which is 10% higher compared to g-C_3_N_4_. Further, the photocatalytic efficiency of Cl/g-C_3_N_4_ was 1.55 times higher than pure g-C_3_N_4_, a fact that has been attributed to the higher active surface area upon chlorination of g-C_3_N_4_.

**Figure 7:**
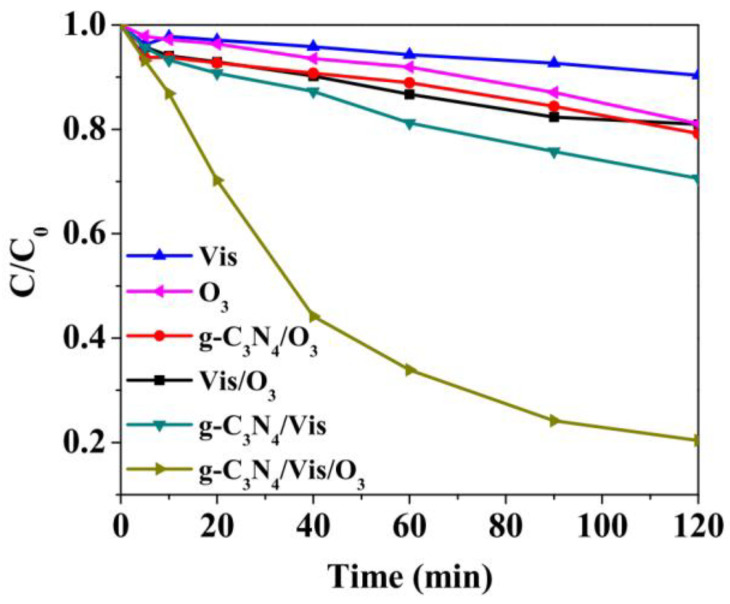
Degradation of OA by different photocatalytic processes. Reproduced with permission from Ref. [58]. Copyright 2014 Elsevier.

**Figure 8:**
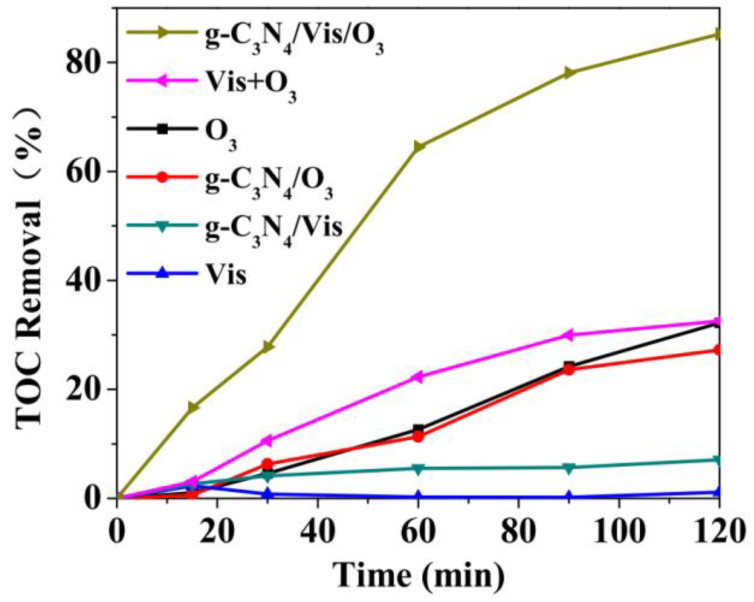
TOC removal of bisphenol A degradation by different processes. Reproduced with permission from Ref. [58]. Copyright 2014 Elsevier.

In one of the earliest reports, g-C_3_N_4_ prepared from thiourea in air at 550 °C was reported as an active photocatalyst for the removal of OA (Figure 7) and bisphenol A (Figure 8) coupling photocatalysis and ozonation.[58] Experimental data showed that OA degradation using g-C_3_N_4_/Vis/O_3_ was 65.2%, which is higher than the sum of the OA degradation values reached by g-C_3_N_4_/Vis and O_3_ separately. The C/C_0_ of g-C_3_N_4_+O_3_ is higher than with g-C_3_N_4_+Vis due to the generation of a high flux of ^•^OH radicals. On the other hand, the TOC removal of bisphenol A with g-C_3_N_4_/Vis/O_3_ was 2.17 times higher than the sum of the ratio with g-C_3_N_4_/Vis and O_3_. This superior performance of the g-C_3_N_4_/Vis/O_3_ system was attributed to the synergistic effect between photocatalysis and ozonation by g-C_3_N_4_. This synergistic effect results in the generation of higher ^•^OH yields, which are the species responsible for the enhanced degradation of organic pollutants.

**Figure 9:**
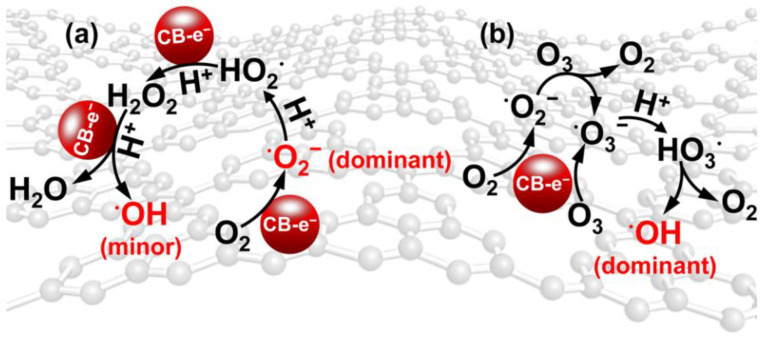
ROS formation pathways in (**a**) Vis/O_2_/C_3_N_4_ and (**b**) Vis/O_3_/C_3_N_4_. Reproduced with permission from Ref. [59]. Copyright 2017 American Chemical Society.

As commented earlier, C_3_N_4_ has been effectively employed as heterogeneous photocatalysts for water disinfection under visible-light irradiation [59]. However, one of the issues that needs to be addressed is the stability of C_3_N_4_ under photocatalytic conditions since the reaction process generates ROS and other reactive radical species. In this aspect, Cao and coworkers have reported the chemical stability of C_3_N_4_ under exposure to ROS during photocatalytic water treatment (Figure 9). The experimental results indicated that ^•^OH can attack the photocatalyst removing the heptazine unit from the C_3_N_4_ sheets, generating secondary pollutants in the aqueous environment. In contrast, C_3_N_4_ is chemically stable toward ^•^O_2_^−^ and O_3_. Interestingly, the decomposition of C_3_N_4_ was fully or partially inhibited in the presence of organic pollutants due to their competition for ^•^OH. Hence, the photocatalyst exhibited high activity and stability under these conditions. This work provides useful information about the chemical instability of C_3_N_4_-based materials in those processes where ^•^OH is the major involved ROS in various applications such as water treatment and organic synthesis.

3. p-Hydroxybenzoic Acid (PHBA)

**Figure 10:**
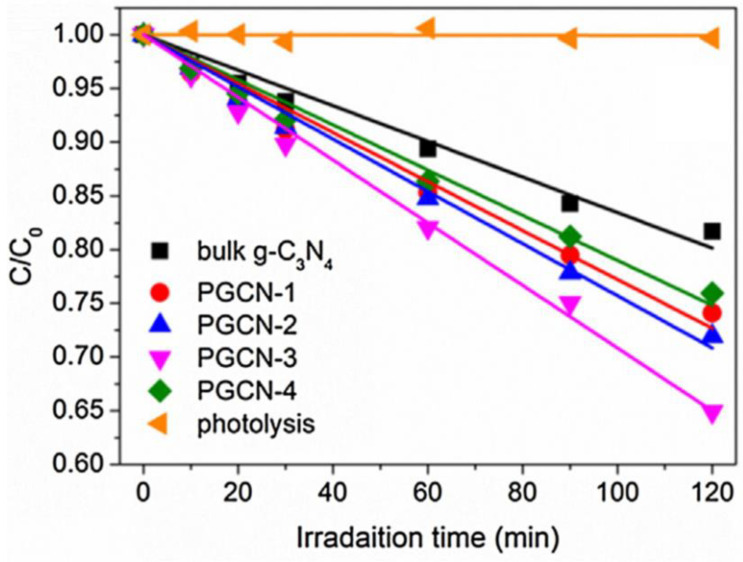
Photocatalytic degradation of PHBA upon irradiation in the absence or presence of different graphitic carbon nitride photocatalysts. Reproduced with permission from Ref. [62]. Copyright 2016 Elsevier.

**Figure 11:**
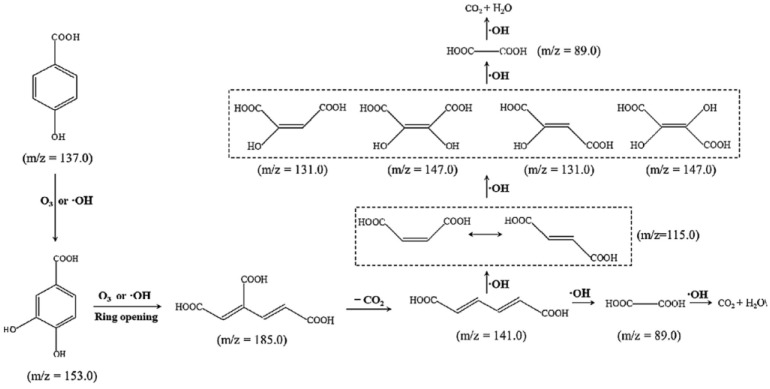
Proposed mechanism for PHBA mineralization in Vis/O_3_/PGCN-3. Reproduced with permission from Ref. [62]. Copyright 2016 Elsevier.

Porous g-C_3_N_4_ (PGCN) has recently received wide attention due to the easy access to the interior of the nanoporous framework [60,61]. In this aspect, a one-pot template-free approach was employed to obtain honeycomb-like PGCN by the reaction between ammonium chloride and the precursor of g-C_3_N_4_, followed by calcination. The photocatalytic activity of PGCN was examined in the photocatalytic activity for PHBA degradation upon visible-light irradiation (Figure 10) [62]. However, PHBA was difficult to mineralize by PGCN as a photocatalyst; an unfavorable factor was the larger band gap of PGCN compared to g-C_3_N_4_. To overcome these difficulties, the photocatalytic activity of PGCN was coupled with ozonation in a Vis/PGCN/O_3_ AOP. The photoactivity data show that photocatalysis by PGCN and O_3_ establish a synergistic effect. Under optimized AOP conditions, Vis/O_3_/PGCN promotes quantitative PHBA mineralization with the dosage of O_3_ as 1.5 mg/min. The process is further accelerated by increasing the O_3_ dosage. This synergism derives from the enhanced generation of ^•^OH. These generated ^•^OH radicals spontaneously react with PHBA and its O_3_-recalcitrant intermediates, such as carboxylic acids, leading to complete mineralization to CO_2_ and H_2_O (Figure 11). This is a nice example illustrating the possible integration of sunlight/PGCN with O_3_ as a metal-free photocatalyst for the AOP in water disinfection.

## 4. Ciprofloxacin

**Figure 12:**
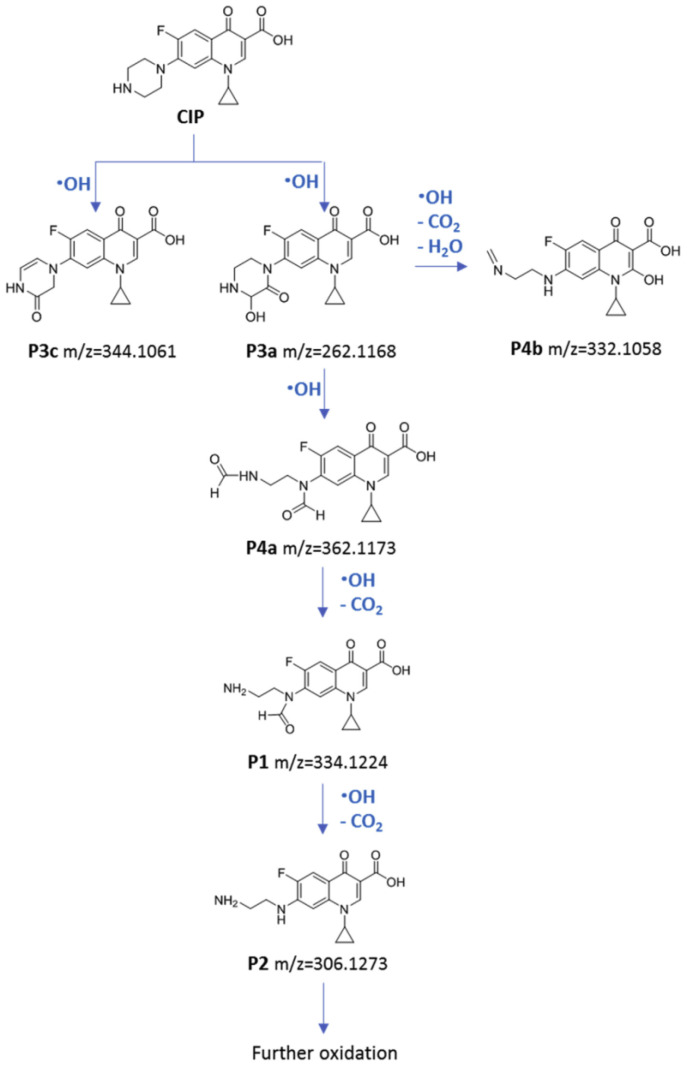
Proposed CIP degradation pathway in the visible-light photocatalytic oxidation with g-C_3_N_4_ nanosheets, based on the detection of intermediate decomposition byproducts. Reproduced with permission from Ref. [63]. Copyright 2020 Elsevier.

In another report, the photocatalytic degradation of a ciprofloxacin (CIP) antibiotic in water was performed using nanosheets of g-C_3_N_4_ as catalysts under visible-light irradiation using white light LEDs. [63] The degradation of CIP was around 90% in 60 min using g-C_3_N_4_ under ozonation conditions using visible-light irradiation. Further, the other objective of this work was the identification of the intermediate byproducts formed upon degradation and to establish the sequential pathway for CIP degradation with possible experimental evidence from liquid chromatography coupled to high-resolution mass spectrometry using a Q-TOF instrument. Seven intermediates were proposed, three of them reported for the first time. Kinetic studies showed that CIP degradation proceeds through a pseudo-first order kinetics with a rate constant of 0.035 min^−1^. The addition of triethanolamine significantly decreased the rate constant to 0.00072 min^−1^, suggesting that CIP degradation is initiated by the holes generated in the catalyst. In addition, the main pathway for CIP degradation was the attack to the piperazine ring by ^•^OH radicals, followed by the rupture of the heterocyclic ring and a suite of consecutive reactions, including the loss of two carbon atoms as CO_2_, defluorination, oxidation and cleavage of the cycles of this intermediate. Figure 12 summarizes the proposed CIP degradation sequence.

## 5. Oxamic Acid (OMA)

**Figure 13:**
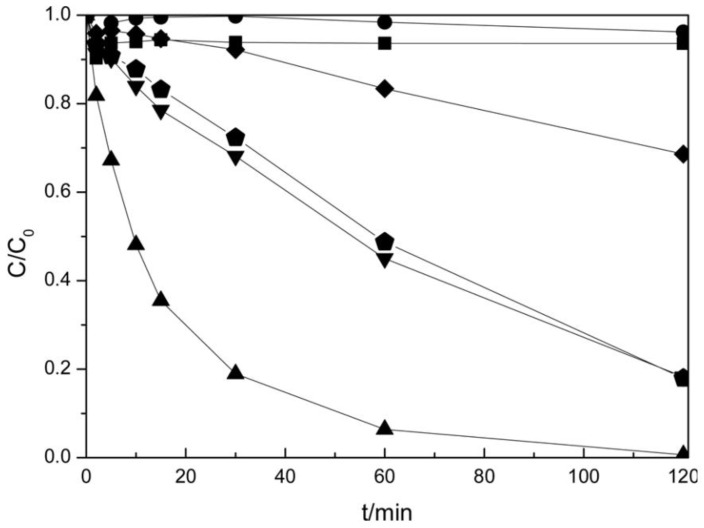
OMA removal by ozonation (◼), photolysis (●), photochemical ozonation (♦), and photocatalytic ozonation using g-C_3_N_4_ (▼), g-C_3_N_4_-500 (▲) and TiO_2_ (⬟). Reproduced with permission from Ref. [64]. Copyright 2020 Elsevier.

Recently, g-C_3_N_4_ has been reported as a heterogeneous photocatalyst for the photocatalytic ozonation of OMA in aqueous solution. The bulk g-C_3_N_4_ material was thermally post-treated at 500 °C to obtain g-C_3_N_4_-500 that exhibits an increased surface area respect to the bulk material [64]. Experimental data show that the photocatalytic ozonation by C_3_N_4_ was highly effective in the removal of OMA, reaching complete OMA degradation with C_3_N_4_-500 after 120 min of irradiation (Figure 13). The high activity of C_3_N_4_-500 is due to the combination of photoinduced charge separation along with ozonation to produce a higher number of ^•^OH radicals. On the other hand, the decrease in the rate of OMA removal in the presence of scavengers is compatible with photogenerated holes on the catalyst surface, playing a dominant role in OMA degradation in comparison to ^•^OH radicals. Although the solid was reused for three cycles without much change in its physicochemical properties, a slight decay in the degradation performance was observed and attributed to modifications in the C_3_N_4_-500 exposed structure occurring in the course of the photocatalytic reaction.

## 6. Parabens

**Figure 14:**
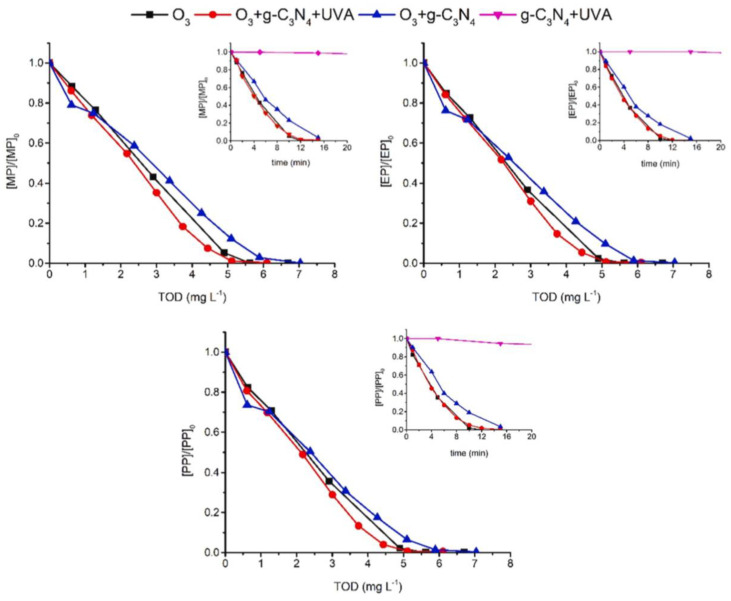
Degradation of methyl-, ethyl- and propylparaben over photocatalytic oxidation and photocatalytic, catalytic, and single ozonation process. Reproduced with permission from Ref. [65]. Copyright 2022 Elsevier.

Besides the above discussed examples with visible-light irradiation, g-C_3_N_4_ was reported as a cost-effective and efficient photocatalyst for the degradation of a mixture of parabens through photo-assisted processes [65]. Control experiments indicated that the use of UV-A radiation exhibited higher activation of g-C_3_N_4_ compared to visible light. The photocatalytic ozonation process showed higher degradation rates of parabens with a ozone dosage lower than the corresponding dark ozonation process. Optimization studies revealed that the medium with basic and neutral conditions (pH = 7–11) provides a better interaction between catalysts and contaminants as well as the highest generation of radicals. Under the optimized reaction conditions of a 500 mg L^−1^ catalyst concentration and a paraben concentration of 1 mg L^−1^, >95% removal was achieved for the three parabens (methyl-, ethyl- and propylparaben) in less than 15 min (Figure 14). Further, these conditions were also effective for the degradation of Allivibrio fischeri bacteria by a significant decrease in its luminescence inhibition, providing a non-toxic, disinfected solution.

The Table 1 summarizes the evidence for the generation of ^•^OH formation and their quantification methods for the various catalysts that have been discussed in this review.

## 7. Conclusions and Prospects

The examples discussed above refer to the degradation of probe molecules by combining photocatalysis by g-C_3_N_4_ and O_3_. Activity data have shown the involvement of synergistic effects by this combination as the AOP, resulting in a degradation level that is much higher than the sum of the degradation degree reached independently by any of the two components. The available mechanistic data indicate that this synergy derives from the higher efficiency of ^•^OH formation with the combined g-C_3_N_4_ photocatalysis/ozonation process due to the capture of the photogenerated electrons in the g-C_3_N_4_ semiconductor by O_3_ as electron acceptor. It has also been commented that, although g-C_3_N_4_ undergoes self-attack by photogenerated ^•^OH, releasing some additional pollutant in water, self-degradation is a minor process when there are some organic molecules present competing for ^•^OH attack. In that way, together with the absence of any transition metal, the combination of the g-C_3_N_4_ photocatalyst and ozonation appears as a practical method, easy to implement for wastewater treatment. Feasibility of implementation also derives from the commercially available large-scale ozonizers that have been already deployed in many plants for wastewater treatment and, thus, a burst in the efficiency can be easily anticipated just by complementing these ozonizers with natural sunlight photocatalysis. The target in this field will be just to confirm laboratory data with probe molecules in real wastewater treatment plants.

## Figures and Tables

**Table 1:** Summary of the of detection of ^•^OH radicals and their quantification by g-C_3_N_4_ catalysts under visible-light irradiation with O_3_.

Catalyst	Detection Method	Quantification	Ref.
Vis/O_3_/GCN-D	Trapping experiments with N_2_, t-butanol and p-benzoquinone	-	[54]
Vis/O_2_/C_3_N_4_	DMPO-OH signal evidenced by EPR	17-fold enhancement of •OH formation	[55]
Vis/O_3_/g-C_3_N_4_	Trapping experiment with t-butanol and the detection of DMPO-OH by EPR	-	[56]
Vis/O_3_/Cl/g-C_3_N_4_	Trapping experiments with N_2_, t-butanol and p-benzoquinone	-	[57]
g-C_3_N_4_/Vis/O_3_	Trapping experiments with t-butanol and triethanolamine	-	[58]
Vis/O_3_/C_3_N_4_	DMPO-OH signal evidenced by EPR	Vis/O_3_/g-C_3_N_4_ generates 6–18 times more •OH	[59]
Vis/O_3_/PGCN	DMPO-OH signal evidenced by EPR	-	[62]
Vis/O_3_/C_3_N_4_ nanosheets	Trapping experiments with t-butanol and triethanolamine	-	[63]
Vis/O_3_/C_3_N_4_-500	Trapping experiments with t-butanol and ethylenediaminetetraacetic acid	-	[64]
O_3_/g-C_3_N_4_/UV-A	-	-	[65]

## References

[1] Chong M.N., Jin B., Chow C.W.K., Saint C. (2010). Recent developments in photocatalytic water treatment technology: A review. Water Res..

[2] Duan X., Sun H., Wang S. (2018). Metal-Free Carbocatalysis in Advanced Oxidation Reactions. ACC Chem. Res..

[3] Kasprzyk-Hordern B., Ziółek M., Nawrocki J. (2003). Catalytic ozonation and methods of enhancing molecular ozone reactions in water treatment. Appl. Catal. B Environ..

[4] Malato S., Fernández-Ibáñez P., Maldonado M.I., Blanco J., Gernjak W. (2009). Decontamination and disinfection of water by solar photocatalysis: Recent overview and trends. Catal. Today.

[5] Martínez-Huitle C.A., Brillas E. (2009). Decontamination of wastewaters containing synthetic organic dyes by electrochemical methods: A general review. Appl. Catal. B Environ..

[6] Nawrocki J., Kasprzyk-Hordern B. (2010). The efficiency and mechanisms of catalytic ozonation. Appl. Catal. B Environ..

[7] Neyens E., Baeyens J. (2003). A review of classic Fenton’s peroxidation as an advanced oxidation technique. J. Hazard. Mater..

[8] Pera-Titus M., García-Molina V., Baños M.A., Giménez J., Esplugas S. (2004). Degradation of chlorophenols by means of advanced oxidation processes: A general review. Appl. Catal. B Environ..

[9] Rivera-Utrilla J., Sánchez-Polo M., Ferro-García M.Á., Prados-Joya G., Ocampo-Pérez R. (2013). Pharmaceuticals as emerging contaminants and their removal from water. A review. Chemosphere.

[10] Wang J.L., Xu L.J. (2012). Advanced oxidation processes for wastewater treatment: Formation of hydroxyl radical and application. Crit. Rev. Environ. Sci. Technol..

[11] Litter M.I., Quici N. (2010). Photochemical advanced oxidation processes for water and wastewater treatment. Recent Pat. Eng..

[12] Nosaka Y., Nosaka A.Y. (2017). Generation and Detection of Reactive Oxygen Species in Photocatalysis. Chem. Rev..

[13] Wang J., Wang S. (2020). Reactive species in advanced oxidation processes: Formation, identification and reaction mechanism. Chem. Eng. J..

[14] Buffle M.-O., Schumacher J., Meylan S., Jekel M., Von Gunten U. (2006). Ozonation and advanced oxidation of wastewater: Effect of O_3_ dose, pH, DOM and HO.-scavengers on ozone decomposition and HO. generation. Ozone Sci. Eng..

[15] Jans U., Hoigné J. (1998). Activated carbon and carbon black catalyzed transformation of aqueous ozone into OH-radicals. Ozone Sci. Eng..

[16] Wu J.J., Wu C.-C., Ma H.-W., Chang C.-C. (2004). Treatment of landfill leachate by ozone-based advanced oxidation processes. Chemosphere.

[17] Chin A., Bérubé P.R. (2005). Removal of disinfection by-product precursors with ozone-UV advanced oxidation process. Water Res..

[18] Javier Benitez F., Acero J.L., Real F.J. (2002). Degradation of carbofuran by using ozone, UV radiation and advanced oxidation processes. J. Hazard. Mater..

[19] Kusic H., Koprivanac N., Bozic A.L. (2006). Minimization of organic pollutant content in aqueous solution by means of AOPs: UV- and ozone-based technologies. Chem. Eng. J..

[20] Agustina T.E., Ang H.M., Vareek V.K. (2005). A review of synergistic effect of photocatalysis and ozonation on wastewater treatment. J. Photochem. Photobiol. C.

[21] Chávez A.M., Rey A., Beltrán F.J., Álvarez P.M. (2016). Solar photo-ozonation: A novel treatment method for the degradation of water pollutants. J. Hazard. Mater..

[22] Xiao J., Xie Y., Rabeah J., Brückner A., Cao H. (2020). Visible-Light Photocatalytic Ozonation Using Graphitic C_3_N_4_ Catalysts: A Hydroxyl Radical Manufacturer for Wastewater Treatment. Acc. Chem. Res..

[23] Rodríguez E.M., Rey A., Mena E., Beltrán F.J. (2019). Application of solar photocatalytic ozonation in water treatment using supported TiO_2_. Appl. Catal. B Environ..

[24] Thomas A., Fischer A., Goettmann F., Antonietti M., Mueller J., Schloegl R., Carlsson J.M. (2008). Graphitic carbon nitride materials: Variation of structure and morphology and their use as metal-free catalysts. J. Mater. Chem..

[25] Wang Y., Wang X., Antonietti M. (2012). Polymeric graphitic carbon nitride as a heterogeneous organocatalyst: From photochemistry to multipurpose catalysis to sustainable chemistry. Angew. Chem. Int. Ed..

[26] Cao S., Low J., Yu J., Jaroniec M. (2015). Polymeric photocatalysts based on graphitic carbon nitride. Adv. Mater..

[27] Cao S., Yu J. (2014). g-C_3_N_4_-based photocatalysts for hydrogen generation. J. Phys. Chem. Lett..

[28] Komatsu T. (2001). Attempted chemical synthesis of graphite-like carbon nitride. J. Mater. Chem..

[29] Kuriki R., Sekizawa K., Ishitani O., Maeda K. (2015). Visible-light-driven CO_2_ reduction with carbon nitride: Enhancing the activity of ruthenium catalysts. Angew. Chem. Int. Ed..

[30] Wen J., Xie J., Chen X., Li X. (2017). A review on g-C_3_N_4_-based photocatalysts. Appl. Surf. Sci..

[31] Yin S., Han J., Zhou T., Xu R. (2015). Recent progress in g-C_3_N_4_ based low cost photocatalytic system: Activity enhancement and emerging applications. Catal. Sci. Technol..

[32] Dong X., Cheng F. (2015). Recent development in exfoliated two-dimensional g-C_3_N_4_ nanosheets for photocatalytic applications. J. Mater. Chem..

[33] Zhang G., Lan Z., Wang X. (2016). Conjugated polymers: Catalysts for photocatalytic hydrogen evolution. Angew. Chem. Int. Ed..

[34] Wang X., Hu W., Wang S., Cai J., Zhang L., Dong L., Zhao L., He Y. (2014). Synthesis and photocatalytic activity of SiO_2_/g-C_3_N_4_ composite photocatalyst. Mater. Lett..

[35] Zhang J., Zhang G., Chen X., Lin S., Moehlmann L., Dolega G., Lipner G., Antonietti M., Blechert S., Wang X. (2012). Co-monomer control of carbon nitride semiconductors to optimize hydrogen evolution with visible light. Angew. Chem. Int. Ed..

[36] Gu Y.L., Chen L.Y., Shi L., Ma J.H., Yang Z.H., Qian Y.T. (2003). Synthesis of C_3_N_4_ and graphite by reacting cyanuric chloride with calcium cyanamide. Carbon.

[37] Zimmerman J.L., Williams R., Khabashesku V.N., Margrave J.L. (2001). Synthesis of spherical carbon nitride nanostructures. Nano Lett..

[38] Xie M., Wei W., Xu Y., Jiang Z., Xie J. (2016). Carbon nitride nanowires/nanofibers: A novel template-free synthesis from a cyanuric chloride-melamine precursor towards enhanced adsorption and visible-light photocatalytic performance. Ceram. Int..

[39] Maeda K., Wang X., Lu D., Nishihara Y., Antonietti M., Domen K. (2009). Photocatalytic activities of graphitic carbon nitride powder for water reduction and oxidation under visible light. J. Phys. Chem. C.

[40] Ji H., Hu X., Chang F., Qin W., Shen J. (2013). Photocatalytic degradation of 2,4,6-trichlorophenol over g-C_3_N_4_ under visible light irradiation. Chem. Eng. J..

[41] Li X., Zhang J., Shen L., Ma Y., Lei W., Cui Q., Zou G. (2009). Preparation and characterization of graphitic carbon nitride through pyrolysis of melamine. Appl. Phys. Mater. Sci. Process..

[42] Cao L., Wang R., Wang D. (2015). Synthesis and characterization of sulfur self-doped gC_3_N_4_ with efficient visible-light photocatalytic activity. Mater. Lett..

[43] Dong F., Wu L., Sun Y., Fu M., Wu Z., Lee S.C. (2011). Efficient synthesis of polymeric gC_3_N_4_ layered materials as novel efficient visible light driven photocatalysts. J. Mater. Chem..

[44] Wang X., Blechert S., Antonietti M. (2012). Polymeric Graphitic Carbon Nitride for Heterogeneous Photocatalysis. ACS Catal..

[45] Gągol M., Przyjazny A., Boczkaj G. (2018). Wastewater treatment by means of advanced oxidation processes based on cavitation—A review. Chem. Eng. J..

[46] Luo H., Fu H., Yin H., Lin Q. (2022). Carbon materials in persulfate-based advanced oxidation processes: The roles and construction of active sites. J. Hazard. Mater..

[47] Zhu X., Zhou Q., Xia Y., Wang J., Chen H., Xu Q., Liu J., Feng W., Chen S. (2021). Preparation and characterization of Cu-doped TiO_2_ nanomaterials with anatase/rutile/brookite triphasic structure and their photocatalytic activity. J. Mater. Sci. Mater. Electron..

[48] Zhu X., Wang J., Yang D., Liu J., He L., Tang M., Feng W., Wu X. (2021). Fabrication, characterization and high photocatalytic activity of Ag–ZnO heterojunctions under UV-visible light. RSC Adv..

[49] Asghar A., Raman A.A.A., Daud W.M.A.W. (2015). Advanced oxidation processes for in-situ production of hydrogen peroxide/hydroxyl radical for textile wastewater treatment: A review. J. Clean. Prod..

[50] Cheng M., Zeng G., Huang D., Lai C., Xu P., Zhang C., Liu Y. (2016). Hydroxyl radicals based advanced oxidation processes (AOPs) for remediation of soils contaminated with organic compounds: A review. Chem. Eng. J..

[51] Ikhlaq A., Brown D.R., Kasprzyk-Hordern B. (2012). Mechanisms of catalytic ozonation on alumina and zeolites in water: Formation of hydroxyl radicals. Appl. Catal. B Environ..

[52] Yang Y., Jiang J., Lu X., Ma J., Liu Y. (2015). Production of Sulfate Radical and Hydroxyl Radical by Reaction of Ozone with Peroxymonosulfate: A Novel Advanced Oxidation Process. Environ. Sci. Technol..

[53] Shinde S.S., Bhosale C.H., Rajpure K.Y., Lee J.H. (2014). Remediation of wastewater: Role of hydroxyl radicals. J. Photochem. Photobiol. B Biol..

[54] Xiao J., Xie Y., Nawaz F., Jin S., Duan F., Li M., Cao H. (2016). Super synergy between photocatalysis and ozonation using bulk g-C_3_N_4_ as catalyst: A potential sunlight/O_3_/g-C_3_N_4_ method for efficient water decontamination. Appl. Catal. B Environ..

[55] Xiao J., Rabeah J., Yang J., Xie Y., Cao H., Brückner A. (2017). Fast Electron Transfer and •OH Formation: Key Features for High Activity in Visible-Light-Driven Ozonation with C_3_N_4_ Catalysts. ACS Catal..

[56] Xiao J., Xie Y., Cao H., Wang Y., Guo Z., Chen Y. (2016). Towards effective design of active nanocarbon materials for integrating visible-light photocatalysis with ozonation. Carbon.

[57] Xiao J., Xie Y., Cao H., Wang Y., Zhao Z. (2015). g-C_3_N_4_-triggered super synergy between photocatalysis and ozonation attributed to promoted •OH generation. Catal. Commun..

[58] Liao G., Zhu D., Li L., Lan B. (2014). Enhanced photocatalytic ozonation of organics by g-C_3_N_4_ under visible light irradiation. J. Hazard. Mater..

[59] Xiao J., Han Q., Xie Y., Yang J., Su Q., Chen Y., Cao H. (2017). Is C_3_N_4_ Chemically Stable toward Reactive Oxygen Species in Sunlight-Driven Water Treatment?. Environ. Sci. Technol..

[60] Zheng Y., Liu J., Liang J., Jaroniec M., Qiao S.Z. (2012). Graphitic carbon nitride materials: Controllable synthesis and applications in fuel cells and photocatalysis. Energy Environ. Sci..

[61] Ran J., Ma T.Y., Gao G., Du X.-W., Qiao S.Z. (2015). Porous P-doped graphitic carbon nitride nanosheets for synergistically enhanced visible-light photocatalytic H_2_ production. Energy Environ. Sci..

[62] Xiao J., Xie Y., Nawaz F., Wang Y., Du P., Cao H. (2016). Dramatic coupling of visible light with ozone on honeycomb-like porous g-C_3_N_4_ towards superior oxidation of water pollutants. Appl. Catal. B Environ..

[63] Jimenez-Salcedo M., Monge M., Teresa Tena M. (2020). Study of intermediate by-products and mechanism of the photocatalytic degradation of ciprofloxacin in water using graphitized carbon nitride nanosheets. Chemosphere.

[64] Orge C.A., Sampaio M.J., Faria J.L., Fernando M., Pereira R., Silva C.G. (2020). Efficiency and stability of metal-free carbon nitride in the photocatalytic ozonation of oxamic acid under visible light. J. Environ. Chem. Eng..

[65] Fernandes E., Drosopoulou S., Mazierski P., Miodynska M., Gołaszewska D., Zaleska-Medynska A., Martins R.C., Gomes J. (2022). Carbon nitride photoactivation evaluation and degradation of a mixture of parabens by ozone assistance. J. Water Proc. Eng..

